# Hierarchical Nitrogen-Doped Porous Carbon Microspheres as Anode for High Performance Sodium Ion Batteries

**DOI:** 10.3389/fchem.2019.00733

**Published:** 2019-10-31

**Authors:** Kaiqi Xu, Qicang Pan, Fenghua Zheng, Guobin Zhong, Chao Wang, Shijia Wu, Chenghao Yang

**Affiliations:** ^1^Electric Power Research Institute of Guangdong Power Grid Co., Ltd., Guangzhou, China; ^2^Guangzhou Key Laboratory for Surface Chemistry of Energy Materials, New Energy Research Institute, School of Environment and Energy, South China University of Technology, Guangzhou, China

**Keywords:** Ni-MOF, hierarchical porosity, carbon microspheres, anode material, sodium ion batteries

## Abstract

Sodium ion batteries (SIBs) have been considered as a promising alternative to lithium ion batteries (LIBs) for large scale energy storage in the future. However, the commercial graphite anode is not suitable for SIBs because of its low Na^+^ ions storage capability and poor cycling stability. Recently, another alternative as anode for SIBs, amorphous carbon materials, have attracted tremendous attention because of their abundant resource, nontoxicity, and most importantly, stability. Here, N-doped hierarchical porous carbon microspheres (NHPCS) derived from Ni-MOF have been prepared and used as anode for SIBs. Benefiting from the open porous structure and expanded interlayer distance, the diffusion of Na^+^ is greatly facilitated and the Na^+^ storage capacity is significantly enhanced concurrently. The NHPCS exhibit high reversible capacity (291 mA h g^−1^ at current of 200 mA g^−1^), excellent rate performance (256 mA h g^−1^ at high current of 1,000 mA g^−1^), and outstanding cycling stability (204 mA h g^−1^ after 200 cycles).

## Introduction

Lithium ion batteries (LIBs) have been widely used as the power sources for portable electric devices, electric vehicles (EV), and hybrid electric vehicles (HEVs), because of their high energy density, high operate voltage and long services life (Zheng et al., 2015; Huang et al., 2016; Wang et al., 2017; Pan et al., 2018a). While, the applications LIBs in large-scale electrochemical energy system (EES) is restricted by the limited lithium resources in the earth's crust (Liu Y. Z. et al., 2018). Alternatively, sodium ion batteries (SIBs) working with the similar intercalation chemistry have attracted much attention, for the abundance of Na on the earth (Larcher and Tarascon, 2015; Li W. et al., 2015; Li et al., 2018). Nevertheless, the commercial graphite anode is not suitable for the fabrication of high performance SIBs, because of the poor diffusion kinetics of the much larger Na^+^ ions compared to Li^+^ ions (Luo et al., 2016; Hwang et al., 2017). Therefore, it is urgent to find an appropriate anode material for SIBs.

Recently, metallic alloy, metal sulfide and metal oxide have been explored as anode materials for SIBs (Liu et al., 2015; Ning et al., 2015; Pan et al., 2018b). Although exhibiting high capacity, these materials are subject to large volume change during the sodiation/desodiation process, and as a result, they are reported to suffer fast capacity loss and poor rate capability (Yabuuchi et al., 2014). Carbonaceous materials, for example, expanded graphite, graphene, carbon nanotubes, and other amorphous carbon, were also studied as alternatives (Wen et al., 2014; Luo et al., 2015; Xu et al., 2015; Li et al., 2016a). These materials have attracted many attentions because of their abundant resource, stability, and low cost (Hou et al., 2017a). Among them, amorphous carbon, e.g., hard carbon and soft carbon, show high Na^+^ accommodation capacity (Li et al., 2016b; Jian et al., 2017), but low initial coulombic efficiency, low capacity, and fast capacity degradation (Qian et al., 2018).

Heteroatom-doping with B, N, S, or P is an effective strategy to enhance the electrical conductivity and increase the specific surface area carbonaceous materials, hence enhance electron transport and provide more Na^+^ storage active sites (Li D. et al., 2015; Yang et al., 2015). P-doped carbon nanosheets, S-doped hard carbon, N-doped carbon, and S, N-codoped carbon nanosheets have been demonstrated to effectively improve the electrochemical performance (Wang et al., 2016; Hou et al., 2017b; Yang et al., 2017; Hong et al., 2018). Hollow porous structures have promise to improve the cycling and rate performance of carbonaceous materials for SIBs, as this unique porous structure (1) can offer more Na^+^ storage active sites, and therefore has the potential to give rise to higher capacity; (2) offer faster Na^+^ transportation channels, which contributes to excellent rate capability; and (3) buffer the volume expansion during the repeated sodiation/desodiation process, leading to improved cycling performance (Li Z. et al., 2015;Zhang et al., 2015).

Herein, we developed a synergetic strategy to combine the above-mentioned merits of the heteroatom-doping and porous structures into one material by preparing Ni-MOF derived N-doped hierarchical porous carbon microspheres (NHPCS). This hierarchical porous structure are shown to offer more active sites for Na^+^ storage, provide fast Na^+^ diffusion channels, and alleviate the volume expansion during the sodiation/desodiation process. Meanwhile, the incorporation of N expanded interlayer distance and increased the surface area of the carbon microspheres, which enhances the cycling and rate performance. Benefiting from this unique design, NHPCS combines the advantages of both heteroatom-doping and porous structures, and are shown to exhibit high reversible capacity, outstanding rate performance and good cycling stability.

## Experimental Section

0.864 g nickel nitrate hexahydrate, 0.3 g 1, 3, 5-benzenetri-carboxylic acid (H3BTC), and 3.0 g polyvinylpyrrolidone (PVP) were dissolved in a mixed solution (20 ml DMF, 20 ml absolute ethanol, and 20 ml deionized water, stirring for 30 min). The as-obtained mixture was transferred into 100 mL Teflon-lined autoclave and heated at 150°C for 10 h. Afterwards, the formed participate was centrifuged with ethanol three times and dried at 80°C overnight. Subsequently, the obtained Ni-MOF was annealed with melamine (with a weight ratio of 1:1) at 800°C for 1 h under Ar atmosphere. Finally, the obtained product was wash with 2 M HCl to remove Ni nanoparticles and the N-doped hierarchical porous carbon microspheres (NHPCS) were obtained. Hierarchical porous carbon microspheres (HPCS) were synthesized with the same procedure except that annealing process was conducted without melamine.

## Material Characterization

The crystalline phases of samples were characterized by XRD (Rigaku D/max 2500). The morphology of NHPCS/HPCS were observed by SEM (Hitachi, SU8010) and TEM (JEOL JEM-2100F). The XPS tests were carried out to determine the chemical composition and chemical state of elements of the sample.

To determine the specific area, the nitrogen absorption/desorption isotherm measurements were performed using ASAP2020 Surface Area and Porosity Analyzer. Raman measurements were performed using a laser Raman spectrometer (Jobin Yvon, Model T6400).

## Electrochemical Measurements

To prepare electrode, the NHPCS/HPCS active material was mixed with PVDF and acetylene black with a mass ratio of 8:1:1. N-methyl-2-pyrrolidene (NMP) was added to the mixture and a uniform slurry was obtained after grinding. The slurry was then coated onto the Cu foil, and dried at 80°C for 12 h. Subsequently, 2,032 coin cells were assembled using sodium metal foil as counter electrode, glass fiber as separator. The electrolyte was 1 M NaCF_3_SO_3_ in DEGDME. The cycling and rate performance of NHPCS and HPCS was tested at Land BT2013A systems using CR2032 coin cell.

## Results and Discussions

**Figure 1:**
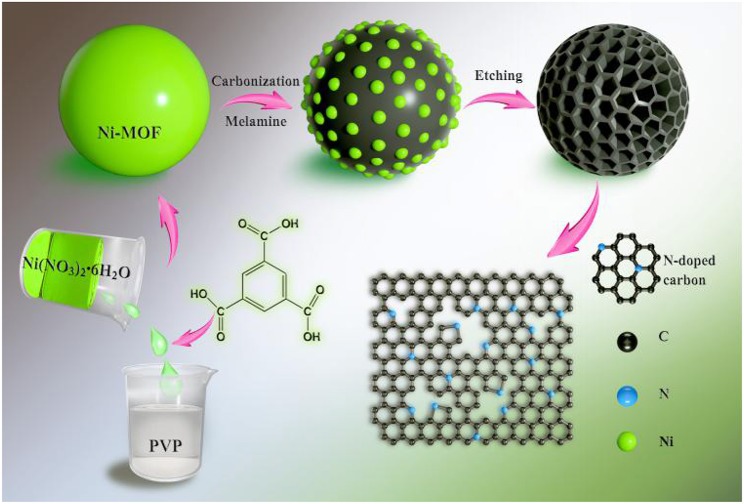
Schematic illustration for the fabrication of N-doped hierarchical porous carbon microspheres (NHPCS).

Figure 1 shows the overall schematic illustration for the fabrication of N-doped hierarchical porous carbon microspheres (NHPCS). Firstly, Ni-MOF precursors were obtained *via* a simple hydrothermal method (see Experimental Section for detail). PVP plays an important role to form the Ni-MOF spheres during the hydrothermal process. The shape inducing effects of PVP could be attributed to the groups of hydrophobic vinyl and hydrophilic carbonyl that lead to the formation of polarized micelles. Subsequently, the obtained Ni-MOF precursors were calcinated with melamine under Ar atmosphere. This annealing process converts MOF to N-C microspheres while maintains the framework of Ni-MOF template, with the Ni nanoparticles uniformly distributing in the carbon microspheres. Finally, the NHPCS was obtained after removing the Ni nanoparticles by etching with HCl aqueous solution.

The morphology of the Ni-MOF, Ni/C, Ni/N-C microspheres was investigated by SEM (Supporting Information). The SEM images in Figures S1A,B show that of Ni-MOF precursor exhibits a very uniform sphere structure with a diameter of around 1.5 μm. This uniform sphere structure was successfully retained after the annealing process, as confirmed from the morphology of the as-formed Ni/C microspheres (Figure S1C) The existence of nickel metal was confirmed by XRD (Figure S2A; Lou et al., 2017). Ni/N-C microspheres was synthesized by annealing the Ni-MOF precursor with melamine. Notably, the uniform sphere structure was well maintained after the N-doping process, as shown in Figure S1D. XRD patterns were collected for HPCS and NHPCS (Figure S2A). A broad peak located at around 24.6° was observed for both samples, which attributed to the (002) diffraction of the disordered carbon structure (Xu et al., 2016; Niu et al., 2017). In the final product, the existence of small amount of Ni nanoparticles might greatly enhance its electronic conductivity.

**Figure 2:**
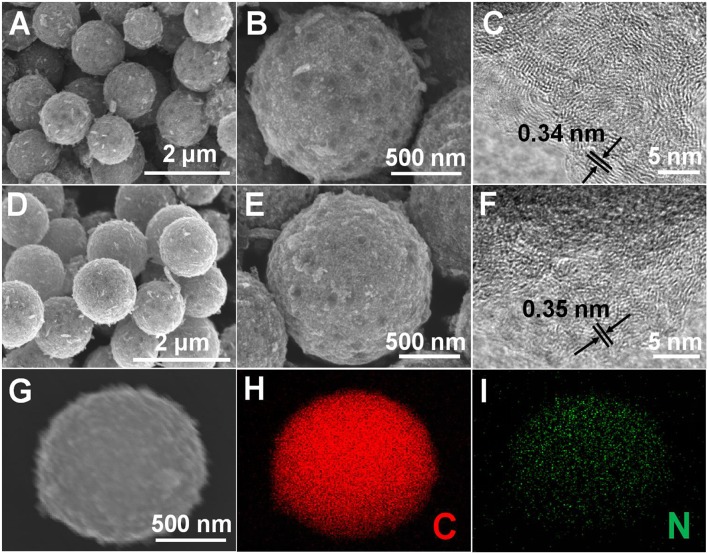
SEM and TEM images of HPCS **(A–C)** and NHPCS **(D–F)**, EDS elemental mapping of NHPCS **(G–I)**.

The structural features of HPCS and NHPCS were examined by SEM and TEM. The SEM results strongly support that the morphology of HPCS and NHPCS are well consistent with the Ni-MOF precursor (Figures 2A,B,D,E), which confirms that the morphology of Ni-MOF precursor can be well maintained during the annealing process. TEM images of HPCS and NHPCS were displayed in Figure S3. There are abundant micropore interconnected with each other in HPCS and NHPCS. On the other hand, minor amount of Ni nanoparticles were retained in the obtained HPCS and NHPCS, which is in good accordance with the XRD results. The HRTEM images exhibit that the interlayer distance of HPCS and NHPCS are 0.34 and 0.35 nm, respectively (Figures 2C,F). This indicates that nitrogen doping can increase the interlayer spacing between the carbon layers (Yan et al., 2016). Moreover, the EDS elemental mapping of NHPCS shows that the N atoms are uniformly dispersed in the carbon microspheres, suggesting that the nitrogen is successfully doped into carbon spheres (Figures 2H,I).

**Figure 3:**
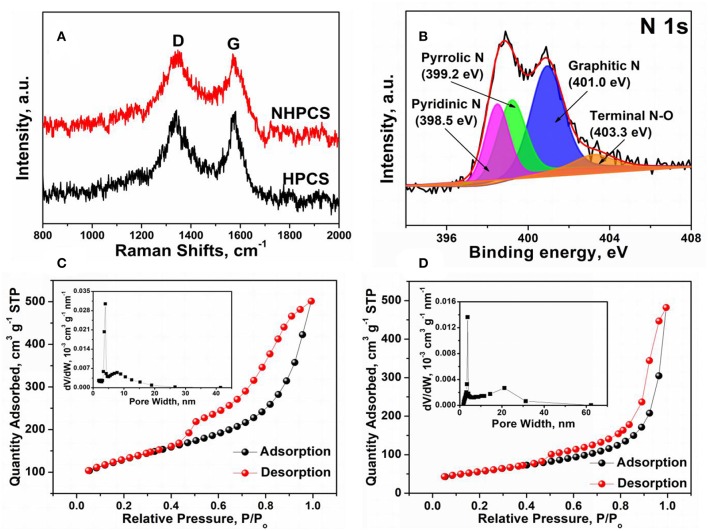
**(A)** Raman spectra of HPCS and NHPCS; **(B)** XPS N 1 s spectra of NHPCS; **(C,D)** N_2_ adsorption isotherm and corresponding pore width distribution for HPCS and NHPCS.

Figure 3A displays the Raman spectra of HPCS and NHPCS. Two peaks are exhibited at around 1,338 and 1,582 cm^−1^, which can be ascribed to the D band and G band corresponding to defect-induced band and crystalline graphite band, respectively (Liu Y. et al., 2018). Therefore, the relative amount of disorder or defects in the carbon structure can be obtained according to the relative intensity ration of the D peak to the G peak (I_D_/I_G_). The I_D_/I_G_ values of HPCS and NHPCS are 1.0 and 1.02, respectively, implying that nitrogen doping improves the disorder degree of carbon materials (Li et al., 2016c).

The XPS measurement was carried out to investigate the surface chemical composition of NHPCS. The survey spectra indicate that C, O, Ni, and N elements exist in NHPCS, which can be further confirmed that the existence of small amount of Ni nanoparticles etching with HCl aqueous solution (Figure S2B). And the high-resolution N 1 s spectra as shown in Figure 3B, four peaks at 398.5, 399.2, 401.0, and 403.3 eV can be ascribed to the typical pyridinic, pyrrolic, graphitic, and quaternary nitrogen, respectively (Liu et al., 2016). The XPS results further confirm that the N-doped carbon was successfully obtained. Furthermore, nitrogen adsorption-desorption isotherms were performed to characterize the surface area and pore structure of HPCS and NHPCS. Figures 3C,D displays the nitrogen adsorption-desorption isotherms for HPCS and NHPCS (inset of C and D are the corresponding pore size distribution curves), respectively. The two curves exhibit similar typical IV isortherm with H_2_-type hysteresis loops at the relative pressure (P/P_0_) range of 0.5–1.0, suggesting the mesoporous features of the structures, and the specific surface area for HPCS and NHPCS are 200.21 and 223.25 m^2^ g^−1^, respectively. The pores of HPCS are mainly distributed at 3.97 and 7.81 nm, while the pores of NHPCS are mainly distributed at 3.72 and 21.15 nm according to the pore size distribution curves, which further suggesting the mesoporous features of the structures in the HPCS and NHPCS.

**Figure 4:**
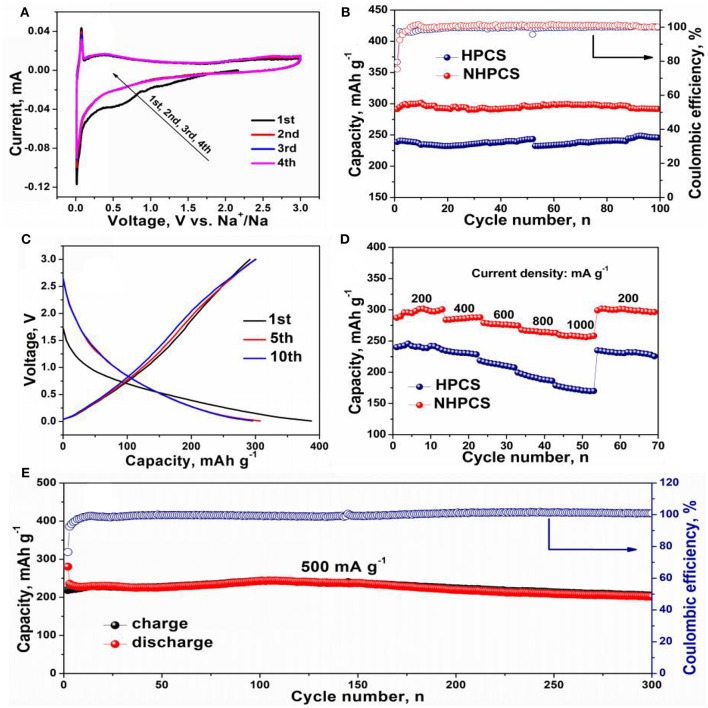
CV curves of NHPCS **(A)**, cycle performance of HPCS and NHPCS at 200 mA g^−1^ **(B)**, charge and discharge profiles of NHPCS **(C)**, rate performance of HPCS and NHPCS **(D)**, long-term cycling performance of NHPCS at 500 mA g^−1^ **(E)**.

The electrochemical performance of HPCS and NHPCS were evaluated using 2,032 coin cells and 1 M NaCF_3_SO_3_ in DEGDME used as the electrolyte. Figure 4A shows the CV curves of NHPCS electrode during the initial four cycles. In the first discharge process, two peaks at around 1.0 and 0.6 V can be observed, which are disappeared in next three cycles, corresponding to the formation of SEI film and side reactions (Hong et al., 2014; Zhu et al., 2018). Subsequently, a strong redox peak at around 0.03 V can be observed, which can be ascribed to Na^+^ insertion/extraction from NHPCS. And the CV curves are highly overlapped with each other after the first cycle, which indicating an outstanding reversible cycling stability.

Figure 4B displays the cycling performance of HPCS and NHPCS at 200 mA g^−1^, a high reversible capacity of 291.8 mA h g^−1^ can be obtained after 100 cycles for NHPCS. And NHPCS also exhibits outstanding cycling stability during the charge/discharge process at 200 mA g^−1^. But for HPCS, lower reversible capacity of 245.9 mA h g^−1^ was obtained after 100 cycles at the same current density. Furthermore, the coulombic efficiency of NHPCS approaches 100% during the cycling process, which is higher than HPCS. Subsequently, the charge and discharge profiles of NHPCS are provided in Figure 4C, a long slope below 1.0 V in the first sodiation process can be observed. For the charge process, the curve became steeper. The initial discharge and charge capacities of NHPCS are 387 and 291 mA h g^−1^, corresponding to an initial coulombic efficiency of 75.2%. And the charge and discharge profiles of HPCS are provided in Figure S4A, which show similar shapes to NHPCS. Furthermore, the initial discharge and charge capacities of HPCS are 297.2 and 239.3 mA h g^−1^, corresponding to an initial coulombic efficiency of 80.2 %. Power density is very important for SIBs in practical application for electric vehicles. Therefore, the rate performances of NHPCS and HPCS at different current density range from 200 to 1,000 mA g^−1^ have been tested (Figure 4D). A high reversible capacity of 298, 285, 276, 265, and 256 mA h g^−1^ at current density of 200, 400, 600, 800, and 1,000 mA g^−1^, respectively. And for HPCS, lower reversible capacity of 240, 231, 214, 192, and 172 mA h g^−1^ at current density of 200, 400, 600, 800, and 1,000 mA g^−1^ were obtained, respectively. These results indicate that high reversible capacity and cycling stability can be attributed to, which can facilitate Na^+^ transport, offer more active sites for Na^+^, and improve the adsorption capacity of Na^+^. Furthermore, long-term cycling performance of NHPCS was further tested at 500 mA g^−1^, and the result is display in Figure 4E. NHPCS exhibits excellent cycling stability and high reversible capacity. 204 mA h g^−1^ can be remained after 300 cycles.

**Figure 5:**
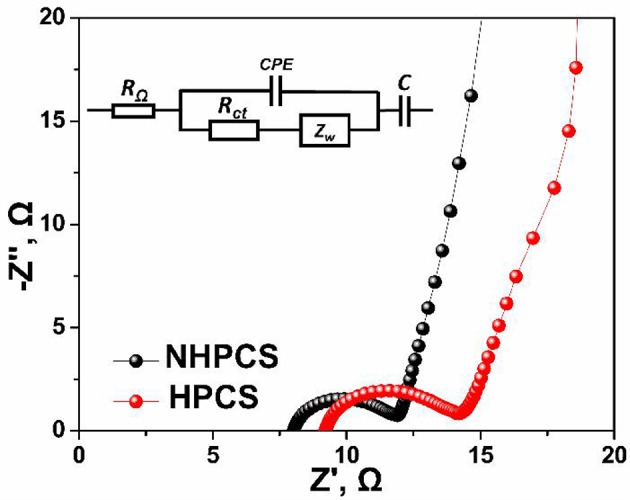
Nyquist plots of NHPCS and HPCS before cycling. Inserted is the equivalent circuit, R_Ω_ is the electrolyte resistance, R_ct_ is the charge-transfer resistance, and Z_w_ is the Warburg impedance.

To further analyze the reasons for the improved cycling and rate performance of NHPCS, the EIS measurements of NHPCS and HPCS were tested, and the corresponding Nyquist plots are shown in Figure 5. The two Nyquist plots exhibit similar shapes and consist of a semicircle in high-frequency region and a sloping line in low-frequency region (Pan et al., 2017; Zheng et al., 2018). Subsequently, charge transfer resistances of NHPCS and HPCS can be obtained according to the EIS curves and equivalent electric circuit (Figure 5). The Rct of NHPCS and HPCS are 3.98 and 5.37Ω, respectively (as shown in Table S1). The lower charge transfer resistance of NHPCS was attributed to the nitrogen doped which can enhance electronic conductivity and facilitate the transport of Na^+^ ions (Wang et al., 2013; Fu et al., 2014). Furthermore, the morphology change after cycling were also investigated to further analyze the outstanding cycling stability of NHPCS. The SEM images of NC after 100 cycles as shown in Figure S6. The sphere structure was maintained well after 100 cycles, indicating that the NHPCS with a good structural stability during the cycling process.

NHPCS exhibits remarkable cycling performance and rate capability when tested as anode for SIBs, which can be owing to the unique hierarchical porous structure and N doping. The detailed reasons can be summarized as follows: (1) The unique hierarchical porous structure can offer more Na^+^ storage active sites and buffer the volume change during the sodiation/desodiation process. (2) The hierarchical porous structure can facilitate Na^+^ transport and provide a short diffusion distance for Na^+^. (3) The nitrogen doping can enhance electronic conductivity to facilitate the transport of Na^+^ and the electrons.

## Conclusions

In summary, N-doped hierarchical porous carbon microspheres (NHPCS) have been successfully fabricated from Ni-MOF with annealed exist of melamine. Benefiting from the nitrogen doped and hierarchical porous structure, NHPCS can offer more Na^+^ storage active sites and provide fast Na^+^ ions transfer channels. As a result, when evaluated as anode for SIBs. NHPCS can exhibit high reversible capacity of 291 mA h g^−1^ at 200 mA g^−1^. When the current density increase to 1,000 mA g^−1^, high reversible capacity of 256 mA h g^−1^ was also can be obtained. Moreover, NHPCS possess a high reversible capacity of 204 mA h g^−1^ after 300 cycles at 500 mA g^−1^. Therefore, NHPCS should be considered as promising anode materials for SIBs because of its outstanding rate capability and excellent cycling performance.

## Data Availability Statement

All datasets generated and analyzed for this study are included in the article/Supplementary Material.

## Author Contributions

KX conducted the experiments. CY is the supervisor of this research work. KX and QP helped with writing. KX, QP, FZ, GZ, CW, and SW performed the characterization and data analysis. All authors involved the analysis of experimental data and manuscript preparation.

### Conflict of Interest

KX, GZ, CW, and SW are now employed by the company Electric Power Research Institute of Guangdong Power Grid Co., Ltd. The authors declare that this study received funding from Electric Power Research Institute of Guangdong Power Grid Co., Ltd. The funder had the following involvement with the study: KX, GZ, and CW were involved in the study design and data analysis. KX and SW were involved in the preparation of the manuscript and decision to publish. The remaining authors declare that the research was conducted in the absence of any commercial or financial relationships that could be construed as a potential conflict of interest.

## References

[B1] Fu L. J. Tang K. Song K. P. van Aken P. A. Yu Y. Maier J. (2014). Nitrogen doped porous carbon fibres as anode materials for sodium ion batteries with excellent rate performance. Nanoscale 6, 1384–1389. 10.1039/c3nr05374a 24306060

[B2] Hong K. L. Qie L. Zeng R. Yi Z. Q. Zhang W. Wang D. (2014). Biomass derived hard carbon used as a high performance anode material for sodium ion batteries. J. Mater. Chem. A 2, 12733–12738. 10.1039/c4ta02068e

[B3] Hong Z. Zhen Y. Ruan Y. Kang M. Zhou K. Zhang J.-M. (2018). Rational design and general synthesis of S-doped hard carbon with tunable doping sites toward excellent Na-ion storage performance. Adv. Mater. 30:1802035 10.1002/adma.201802035 29808566

[B4] Hou H. Qiu X. Wei W. Zhang Y. Ji X. (2017a). Carbon anode materials for advanced sodium-ion batteries. Adv. Energy Mater. 7:1602898 10.1002/aenm.201602898

[B5] Hou H. Shao L. Zhang Y. Zou G. Chen J. Ji X. (2017b). Large-area carbon nanosheets doped with phosphorus: a high-performance anode material for sodium-ion batteries. Adv. Sci. 4:1600243. 10.1002/advs.201600243 28105399 PMC5238737

[B6] Huang Y. G. Pan Q. C. Wang H. Q. Ji C. Wu X. M. He Z. Q. (2016). Preparation of a Sn@SnO_2_@c@MoS_2_ composite as a high-performance anode material for lithium-ion batteries. J. Mater. Chem. A 4, 7185–7189. 10.1039/c6ta02080a

[B7] Hwang J. Y. Myung S. T. Sun Y. K. (2017). Sodium-ion batteries: present and future. Chem. Soc. Rev. 46, 3529–3614. 10.1039/c6cs00776g 28349134

[B8] Jian Z. Bommier C. Luo L. Li Z. Wang W. Wang C. (2017). Insights on the mechanism of Na-ion storage in soft carbon anode. Chem. Mater. 29, 2314–2320. 10.1021/acs.chemmater.6b05474

[B9] Larcher D. Tarascon J. M. (2015). Towards greener and more sustainable batteries for electrical energy storage. Nat. Chem. 7, 19–29. 10.1038/nchem.2085 25515886

[B10] Li B. Dai F. Xiao Q. F. Yang L. Shen J. M. Zhang C. M. (2016c). Nitrogen-doped activated carbon for a high energy hybrid supercapacitor. Energy Environ. Sci. 9, 102–106. 10.1039/c5ee03149d

[B11] Li D. Chen H. Liu G. Wei M. Ding L.-X. Wang S. (2015). Porous nitrogen doped carbon sphere as high performance anode of sodium-ion battery. Carbon 94, 888–894. 10.1016/j.carbon.2015.07.067

[B12] Li L. Zheng Y. Zhang S. L. Yang J. P. Shao Z. P. Guo Z. P. (2018). Recent progress on sodium ion batteries: potential high-performance anodes. Energy Environ. Sci. 11, 2310–2340. 10.1039/c8ee01023d

[B13] Li W. Zhou M. Li H. Wang K. Cheng S. Jiang K. (2015). A high performance sulfur-doped disordered carbon anode for sodium ion batteries. Energy Environ. Sci. 8, 2916–2921. 10.1039/c5ee01985k

[B14] Li Y. Hu Y.-S. Li H. Chen L. Huang X. (2016a). A superior low-cost amorphous carbon anode made from pitch and lignin for sodium-ion batteries. J. Mater. Chem. A 4, 96–104. 10.1039/C5TA08601A

[B15] Li Y. Hu Y. S. Titirici M. M. Chen L. Huang X. (2016b). Hard carbon microtubes made from renewable cotton as high-performance anode material for sodium-ion batteries. Adv. Energy Mater. 6:1600659 10.1002/aenm.201600659

[B16] Li Z. Ding J. Mitlin D. (2015). Tin and Tin compounds for sodium ion battery anodes: phase transformations and performance. Acc. Chem. Res. 48, 1657–1665. 10.1021/acs.accounts.5b00114 26046961

[B17] Liu H. Jia M. Cao B. Chen R. Lv X. Tang R. (2016). Nitrogen-doped carbon/graphene hybrid anode material for sodium-ion batteries with excellent rate capability. J. Power Sources 319, 195–201. 10.1016/j.jpowsour.2016.04.040

[B18] Liu Y. Yang C. Pan Q. Li Y. Wang G. Ou X. (2018). Nitrogen-doped bamboo-like carbon nanotubes as anode material for high performance potassium ion batteries. J. Mater. Chem. A 6, 15162–15169. 10.1039/C8TA04694H

[B19] Liu Y. C. Zhang N. Jiao L. F. Chen J. (2015). Tin nanodots encapsulated in porous nitrogen-doped carbon nanofibers as a free-standing anode for advanced sodium-ion batteries. Adv. Mater. 27, 6702–6707. 10.1002/adma.201503015 26422696

[B20] Liu Y. Z. Zhong W. T. Yang C. H. Pan Q. C. Li Y. P. Wang G. (2018). Direct synthesis of FeS/N-doped carbon composite for high-performance sodium-ion batteries. J. Mater. Chem. A 6, 24702–24708. 10.1039/c8ta08562e

[B21] Lou P. Cui Z. Jia Z. Sun J. Tan Y. Guo X. (2017). Monodispersed carbon-coated cubic NiP_2_ nanoparticles anchored on carbon nanotubes as ultra-long-life anodes for reversible lithium storage. ACS Nano 11, 3705–3715. 10.1021/acsnano.6b08223 28323408

[B22] Luo W. Shen F. Bommier C. Zhu H. L. Ji X. L. Hu L. B. (2016). Na-ion battery anodes: materials and electrochemistry. Acc. Chem. Res. 49, 231–240. 10.1021/acs.accounts.5b00482 26783764

[B23] Luo X.-F. Yang C.-H. Peng Y.-Y. Pu N.-W. Ger M.-D. Hsieh C.-T. (2015). Graphene nanosheets, carbon nanotubes, graphite, and activated carbon as anode materials for sodium-ion batteries. J. Mater. Chem. A 3, 10320–10326. 10.1039/C5TA00727E

[B24] Ning Z. Han X. Liu Y. Hu X. Zhao Q. Chen J. (2015). 3D porous γ-Fe_2_O_3_@C nanocomposite as high-performance anode material of Na-ion batteries. Adv. Energy Mater. 5:1401123 10.1002/aenm.201401123

[B25] Niu J. Liang J. Shao R. Liu M. Dou M. Li Z. (2017). Tremella-like N, O-codoped hierarchically porous carbon nanosheets as high-performance anode materials for high energy and ultrafast Na-ion capacitors. Nano Energy 41, 285–292. 10.1016/j.nanoen.2017.09.041

[B26] Pan Q. C. Zhang Q. B. Zheng F. H. Liu Y. Z. Li Y. P. Ou X.. (2018b). Construction of MoS_2_/C hierarchical tubular heterostructures for high-performance sodium ion batteries. ACS Nano 12, 12578–12586. 10.1021/acsnano.8b07172 30452222

[B27] Pan Q. C. Zheng F. H. Ou X. Yang C. H. Xiong X. H. Liu M. L. (2017). MoS_2_ encapsulated SnO_2_-SnS/C nanosheets as a high performance anode material for lithium ion batteries. Chem. Eng. J. 316, 393–400. 10.1016/j.cej.2017.01.111

[B28] Pan Q. C. Zheng F. H. Wu Y. N. Ou X. Yang C. H. Xiong X. H. (2018a). MoS_2_-covered SnS nanosheets as anode material for lithium-ion batteries with high capacity and long cycle life. J. Mater. Chem. A 6, 592–598. 10.1039/c7ta08346g

[B29] Qian J. Wu F. Ye Y. Zhang M. Huang Y. Xing Y. (2018). Boosting fast sodium storage of a large-scalable carbon anode with an ultralong cycle life. Adv. Energy Mater. 8:1703159 10.1002/aenm.201703159

[B30] Wang H. Q. Pan Q. C. Wu Q. Zhang X. H. Huang Y. G. Lushington A. (2017). Ultrasmall MoS_2_ embedded in carbon nanosheets-coated Sn/SnO_x_ as anode material for high-rate and long life Li-ion batteries. J. Mater. Chem. A 5, 4576–4582. 10.1039/c6ta10932b

[B31] Wang S. Xia L. Yu L. Zhang L. Wang H. Lou X. W. (2016). Free-standing nitrogen-doped carbon nanofiber films: integrated electrodes for sodium-ion batteries with ultralong cycle life and superior rate capability. Adv. Energy Mater. 6:1502217 10.1002/aenm.201502217

[B32] Wang Z. H. Qie L. Yuan L. X. Zhang W. X. Hu X. L. Huang Y. H. (2013). Functionalized N-doped interconnected carbon nanofibers as an anode material for sodium-ion storage with excellent performance. Carbon 55, 328–334. 10.1016/j.carbon.2012.12.072

[B33] Wen Y. He K. Zhu Y. J. Han F. D. Xu Y. H. Matsuda I.. (2014). Expanded graphite as superior anode for sodium-ion batteries. Nat. Commun. 5:4033. 10.1038/ncomms5033 24893716

[B34] Xu D. Chen C. Xie J. Zhang B. Miao L. Cai J. (2016). A hierarchical N/S-codoped carbon anode fabricated facilely from cellulose/polyaniline microspheres for high-performance sodium-ion batteries. Adv. Energy Mater. 6:1501929 10.1002/aenm.201501929

[B35] Xu J. Wang M. Wickramaratne N. P. Jaroniec M. Dou S. Dai L. (2015). High-performance sodium ion batteries based on a 3D anode from nitrogen-doped graphene foams. Adv. Mater. 27, 2042–2048. 10.1002/adma.201405370 25689053

[B36] Yabuuchi N. Kubota K. Dahbi M. Komaba S. (2014). Research development on sodium-ion batteries. Chem. Rev. 114, 11636–11682. 10.1021/cr500192f 25390643

[B37] Yan D. Yu C. Zhang X. Qin W. Lu T. Hu B. (2016). Nitrogen-doped carbon microspheres derived from oatmeal as high capacity and superior long life anode material for sodium ion battery. Electrochim. Acta 191, 385–391. 10.1016/j.electacta.2016.01.105

[B38] Yang F. Zhang Z. Du K. Zhao X. Chen W. Lai Y. (2015). Dopamine derived nitrogen-doped carbon sheets as anode materials for high-performance sodium ion batteries. Carbon 91, 88–95. 10.1016/j.carbon.2015.04.049

[B39] Yang J. Zhou X. Wu D. Zhao X. Zhou Z. (2017). S-doped N-rich carbon nanosheets with expanded interlayer distance as anode materials for sodium-ion batteries. Adv. Mater. 29:1604108. 10.1002/adma.201604108 27885733

[B40] Zhang K. Li X. Liang J. Zhu Y. Hu L. Cheng Q. (2015). Nitrogen-doped porous interconnected double-shelled hollow carbon spheres with high capacity for lithium ion batteries and sodium ion batteries. Electrochim. Acta 155, 174–182. 10.1016/j.electacta.2014.12.108

[B41] Zheng F. H. Ou X. Pan Q. C. Xiong X. H. Yang C. H. Fu Z. Y. (2018). Nanoscale gadolinium doped ceria (GDC) surface modification of Li-rich layered oxide as a high performance cathode material for lithium ion batteries. Chem. Eng. J. 334, 497–507. 10.1016/j.cej.2017.10.050

[B42] Zheng F. H. Yang C. H. Xiong X. H. Xiong J. W. Hu R. Z. Chen Y.. (2015). Nanoscale surface modification of lithium-rich layered-oxide composite cathodes for suppressing voltage fade. Angew. Chem. Int. Ed. 54, 13058–13062. 10.1002/anie.201506408 26335589

[B43] Zhu Z. Y. Liang F. Zhou Z. R. Zeng X. Y. Wang D. Dong P. (2018). Expanded biomass-derived hard carbon with ultrastable performance in sodium-ion batteries. J. Mater. Chem. A 6, 1513–1522. 10.1039/c7ta07951f

