# Supplementary material for: Hierarchical Nitrogen-Doped Porous Carbon Microspheres as Anode for High Performance Sodium Ion Batteries
Source: Front Chem. 2019 Oct 31;7:733. doi: 10.3389/fchem.2019.00733 (PMC6834544; doi:10.3389/fchem.2019.00733)
Supplement: Supplementary file 1 [file Table_1.DOC]

**Supporting Information**

**Hierarchical nitrogen-doped porous carbon microspheres as anode for high performance sodium ion batteries**

Kaiqi Xu^1^, Qicang Pan^2^, Fenghua Zheng^2^, Guobin Zhong^1^, Chao Wang^1^, Shijia Wu^1^, Chenghao Yang2,[[1]](#footnote-2)*

1 Electric Power Research Institute of Guangdong Power Grid Co., Ltd., Guangzhou, Guangdong 510080, China

2 Guangzhou Key Laboratory for Surface Chemistry of Energy Materials, New Energy Research Institute, School of Environment and Energy, South China University of Technology, Guangzhou 510006, P. R. China


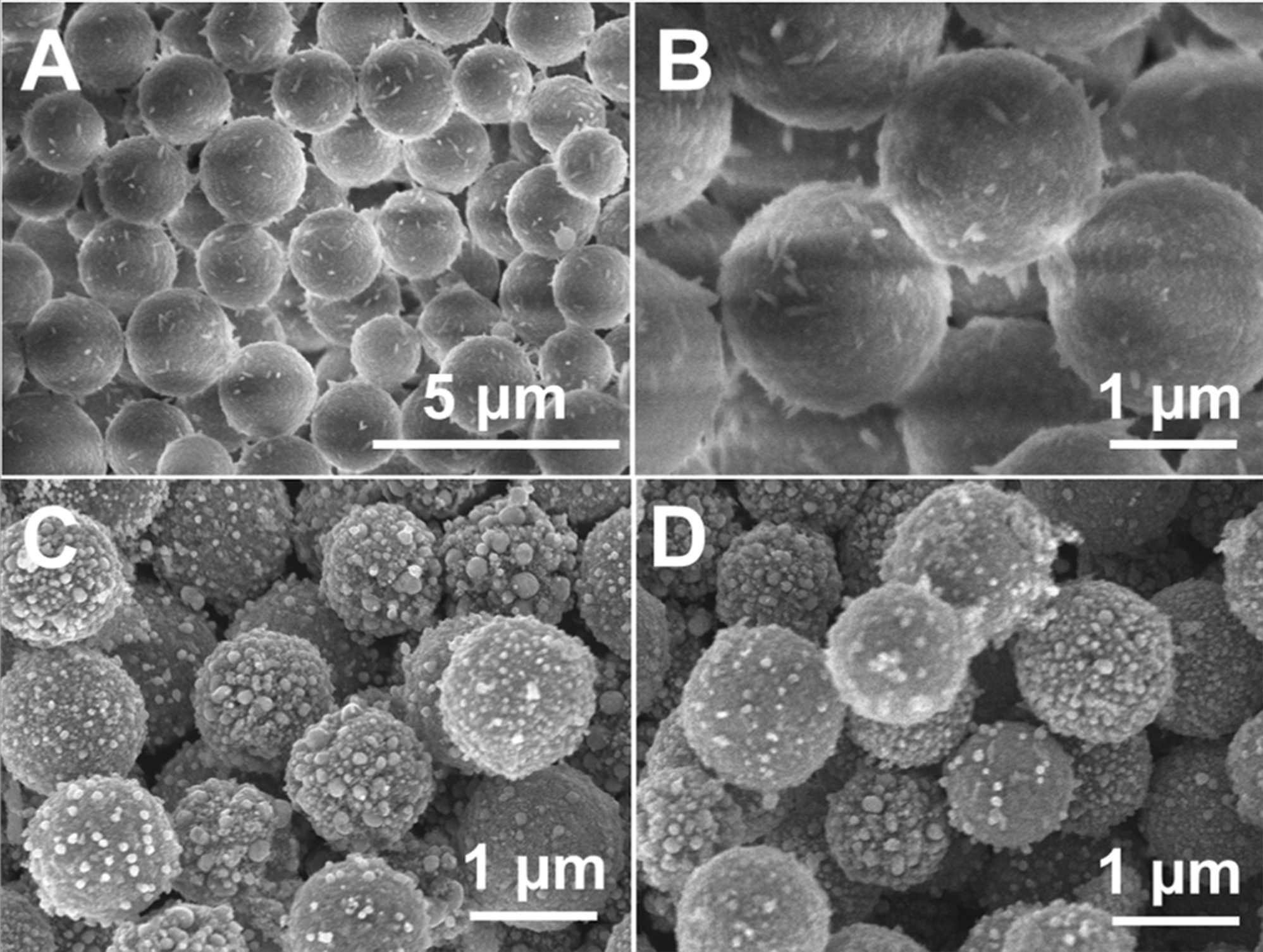


**Fig. S1** SEM images of (A, B) Ni-MOF microspheres, (C) Ni/C and (D) Ni/N-C microspheres.


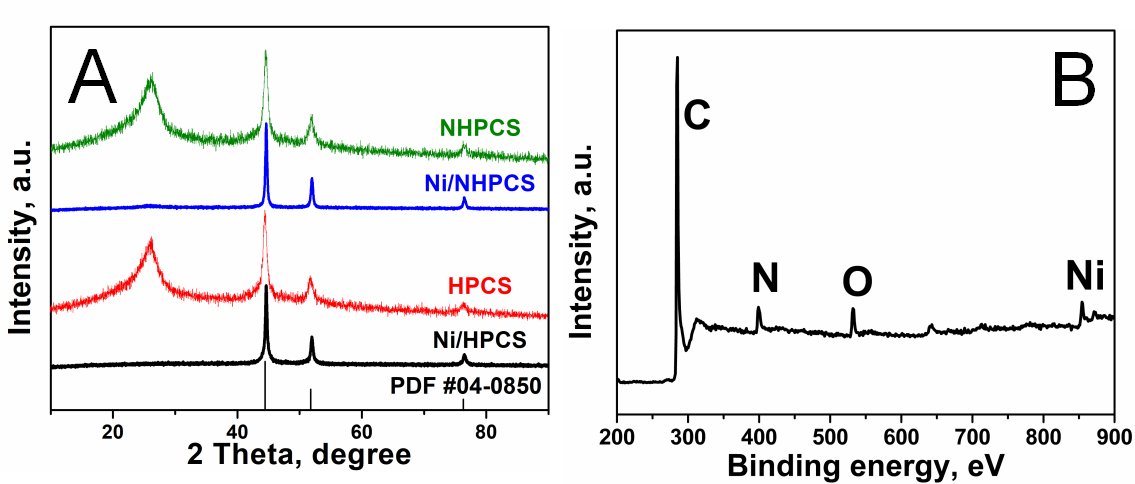


**Fig. S2** XRD patterns of (A) Ni/C, HNCS and NHNCS; (B) XPS spectra of NHNCS.


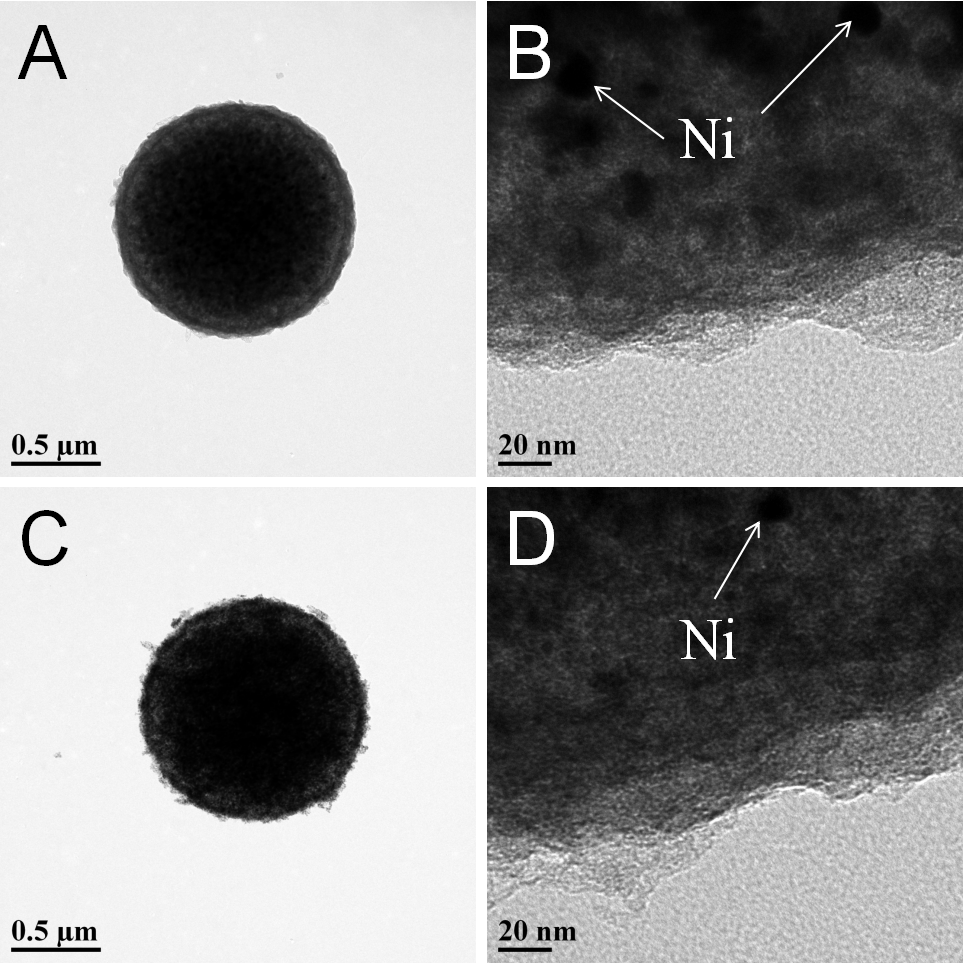


**Fig. S3** TEM images of HNCS (A, B) and NHNCS (C, D).

**
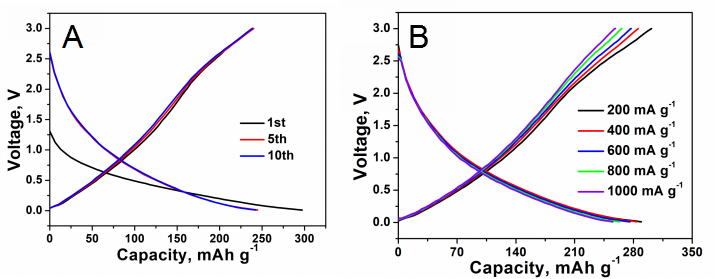
**

**Fig. S4** (A) Charge and discharge profiles of HPCS; (B) Charge and discharge profiles at different charge/discharge rates from 200 mA g^-1^ to 2000 mA g^-1^.

**Table S1** [Impedance parameters](https://www.sciencedirect.com/topics/engineering/impedance-parameter) derived using equivalent [circuit](https://www.sciencedirect.com/topics/materials-science/electronic-circuit) model for as-prepared NHPCS and HPCS.

|  | Rct | R_Ω_ |
| --- | --- | --- |
| NHPCS | 3.98 Ω | 8.0 Ω |
| HPCS | 5.37 Ω | 7.95 Ω |


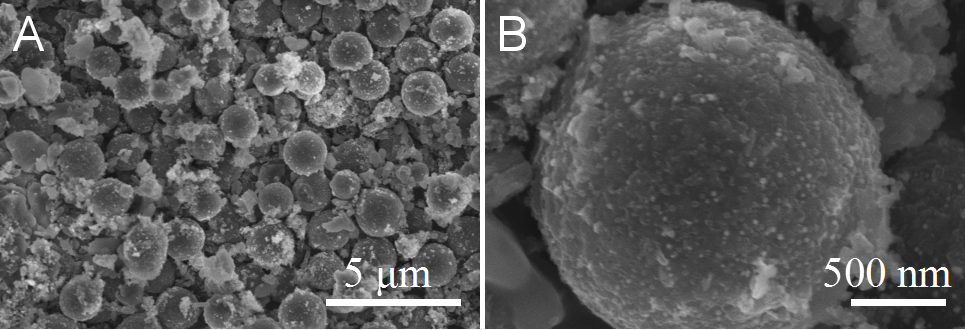


**Fig. S5** SEM images of NHPCS after 100 cycles.

1. *Corresponding author. *E-mail* addresses: esyangc@scut.edu.cn (C. Yang) [↑](#footnote-ref-2)
